# A Broad Assessment of Factors Determining *Culicoides imicola* Abundance: Modelling the Present and Forecasting Its Future in Climate Change Scenarios

**DOI:** 10.1371/journal.pone.0014236

**Published:** 2010-12-06

**Authors:** Pelayo Acevedo, Francisco Ruiz-Fons, Rosa Estrada, Ana Luz Márquez, Miguel Angel Miranda, Christian Gortázar, Javier Lucientes

**Affiliations:** 1 Biogeography, Diversity and Conservation Research Team, Department of Animal Biology, Faculty of Sciences, University of Malaga, Málaga, Spain; 2 Instituto de Investigación en Recursos Cinegéticos IREC (CSIC-UCLM-JCCM), Animal Health Department, Ciudad Real, Spain; 3 Instituto Vasco de Investigación y Desarrollo Agrario Neiker-Tecnalia, Department of Animal Health, Derio, Spain; 4 Department of Animal Pathology, Faculty of Veterinary, University of Zaragoza, Zaragoza, Spain; 5 Department of Biology, University of the Balearic Islands, Palma de Majorca, Spain; University of Leeds, United Kingdom

## Abstract

Bluetongue (BT) is still present in Europe and the introduction of new serotypes from endemic areas in the African continent is a possible threat. *Culicoides imicola* remains one of the most relevant BT vectors in Spain and research on the environmental determinants driving its life cycle is key to preventing and controlling BT. Our aim was to improve our understanding of the biotic and abiotic determinants of *C. imicola* by modelling its present abundance, studying the spatial pattern of predicted abundance in relation to BT outbreaks, and investigating how the predicted current distribution and abundance patterns might change under future (2011–2040) scenarios of climate change according to the Intergovernmental Panel on Climate Change. *C. imicola* abundance data from the bluetongue national surveillance programme were modelled with spatial, topoclimatic, host and soil factors. The influence of these factors was further assessed by variation partitioning procedures. The predicted abundance of *C. imicola* was also projected to a future period. Variation partitioning demonstrated that the pure effect of host and topoclimate factors explained a high percentage (>80%) of the variation. The pure effect of soil followed in importance in explaining the abundance of *C. imicola*. A close link was confirmed between *C. imicola* abundance and BT outbreaks. To the best of our knowledge, this study is the first to consider wild and domestic hosts in predictive modelling for an arthropod vector. The main findings regarding the near future show that there is no evidence to suggest that there will be an important increase in the distribution range of *C. imicola*; this contrasts with an expected increase in abundance in the areas where it is already present in mainland Spain. What may be expected regarding the future scenario for orbiviruses in mainland Spain, is that higher predicted *C. imicola* abundance may significantly change the rate of transmission of orbiviruses.

## Introduction

Understanding the patterns and mechanisms of species occurrence and abundance is a central issue in ecology and epidemiology. Biogeography is an increasingly important discipline and is highly useful for addressing determinants of animal, plant or pathogen species distribution, since the broad-scale factors influencing species spatial patterns can be accurately identified from biogeographical modelling [1]. The increased application of spatial models to conservation biology and wildlife management has been driven, at least in part, by the advent of extensive computerised spatial databases — for example the Worldclim project (www.worldclim.org) — and powerful analytical tools [2].

This discipline has rarely focused on pathogen ecology (but see [3]), i.e. epidemiology [4], as was demonstrated at the 4th meeting of the International Biogeography Society in 2009, where a symposium was designed to introduce to biogeographers the many ways that this discipline can contribute to the study of disease ecology [5]. The scarcity of epidemiological studies conducted from a biogeographical perspective is probably due to the fact that epidemiological processes have key differences compared to other biological phenomena [6]. Thus, abiotic conditions may have less influence on pathogen distribution than on animal or plant species distribution. This is due to the dependence of pathogens on other species, for example hosts, which is even more complex in the case of vector-borne pathogens [7]. Hence, biotic factors in these systems are the main determinants of pathogen distribution, and abiotic factors may indirectly affect their distribution by interfering with host and vector distribution and abundance [8].

In this sense, some studies have been conducted to explain and predict vector distribution and abundance from an epidemiological perspective. Most of them were motivated by disease outbreaks [9], such as bluetongue, malaria or Crimean-Congo hemorrhagic fever [10]–[12]. Nevertheless, a better approach would be to determine the distribution and abundance of vectors in advance of disease outbreaks to establish sound disease risk management policies.

Bluetongue (BT) is one of the vector-borne diseases that has raised increasing interest in vector ecology among epidemiologists. Currently, BT outbreaks are still ongoing in Europe and new introductions remain a risk. An important route of the BT virus (BTV) being introduced into Europe is via wind-borne infected midges arriving from northern African countries. This potential threat should be addressed by research-based protection and prevention policies; in fact, ecological studies on BTV vectors are one of the main targets of scientific preventive research [13]. In this respect, *Culicoides imicola* (Diptera: Ceratopogonidae) is the major vector of BTV in the African continent and Mediterranean Europe [14] where it shares vector ability with other *Culicoides* spp. [15], [16].

*C. imicola* biogeography has already been studied from an epidemiological perspective. These models, based on climate and satellite imagery, identified some of the key parameters determining *C. imicola* distribution in the Mediterranean area. *C. imicola* spatial distribution in Europe is very patchy, showing a high dependence on local habitat conditions, such as soil type, soil moisture and topography [10], [17], [18]. Different *Culicoides* spp. have specialized in using different conditions [19], and breed in a range of moist microhabitats. Nonetheless, soil type strongly determines the ability of *C. imicola* to become established in any given zone, presumably by interfering with the availability of breeding sites [18]–[20]. In South Africa, *C. imicola* has been found to be absent from sandy areas [17], [21], whereas distance from moisture-retentive soil was the most important factor determining *C. imicola* presence in Italy [18]. However, to date, no study has been designed to explore the pure and combined effects of a wide variety of factors (both abiotic and biotic) that determine the distribution and abundance of this arthropod vector species, including the influence of hosts on *C. imicola* population dynamics.

Ever since the initial *C. imicola* distribution models were developed, in which a close link between species distribution and climatic variables was demonstrated, researchers began to assess the effects of climate change on this species [22]. The conventional wisdom was that global climate change would result in an expansion of tropical pathogens, particularly those transmitted by vectors, throughout temperate areas [23], [24]. Despite its high potential relevance, the first approaches conducted on *C. imicola* were quite simplistic — authors considered a putative increase of 2°C in mean temperature and then recalculated the potential species distribution using transformed climatic variables. Nonetheless, projected future scenarios of climate change suggested that global warming would be the main factor modulating the northward expansion of *C. imicola* into Europe [22]. However, the predictions of the climatic models did not perfectly fit the observed abundance data since other factors regulate species distribution (as mentioned) and therefore its expansion [25], [26]. *C. imicola* is able to spread northwards but probably only into those areas where non-climate factors are suitable for the species, as suggested by [10].

In this respect, few statistical models include potentially important non-climate variables. The accuracy of these predictions could be increased by including other ecogeographical variables in the models [27], such as soil characteristics [20], the presence of wild or domestic potential hosts [28] or geographical factors, e.g. showing species population dynamics [29].

In this context, we attempted to improve our understanding of the biotic and abiotic determinants of *C. imicola* as follows: i) by modelling its present abundance with topoclimatic, host, spatial and soil conditions using variation partitioning procedures; ii) by studying the spatial pattern of the predicted abundance of *C. imicola* relative to BT outbreaks to assess the spatial association between vector abundance and BT occurrence; and iii) by investigating how the predicted current distribution and abundance patterns of *C. imicola* might change under future (2011–2040) scenarios of climate change according to the Intergovernmental Panel on Climate Change.

## Materials and Methods

### Study area and *C. imicola* abundance data

The study area was peninsular Spain. This is situated in southwest Europe and covers 493,518 km^2^ (nearly 85% of the Iberian Peninsula). It is a heterogeneous territory in climatic terms, with a mainly eastward and southward decreasing precipitation gradient (range 200–2000 mm) and a mainly northward decreasing temperature gradient [30]. The northern and Mediterranean coasts are bordered by mountain ranges and there are some east-to-west mountain chains in the centre of the Peninsula.

*C. imicola* capture data from 2005 to 2008 were provided by the Spanish bluetongue national surveillance programme; for details see [31]. In line with previous studies [31], only catch data obtained between April and October — annual peak abundance for *C. imicola* in Spain [32] — were used for analytical purposes. The only localities included in the analyses were those where sampling was performed at least once a month between April and October. For each sampled locality we obtained the maximum number of *C. imicola* captured per night during that period (April–October) and during the 3 years considered in this study as this abundance index (our response variable) has been shown to be consistently related to the real *Culicoides* spp. annual abundance [33].

**Figure 1:**
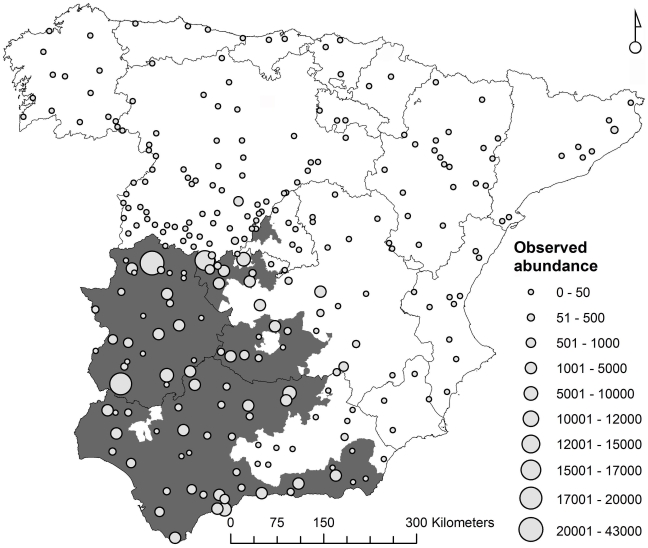
Location of the sampling localities. Spatial distribution of the sites (UTM 10×10 km squares) in which the abundance of *Culicoides imicola* was recorded (n = 263) by the Spanish national bluetongue surveillance programme between 2005 and 2008 (symbol size is proportional to the maximum number of *C. imicola* captures per night). These were used as a training dataset to forecast the species abundance in peninsular Spain. Regional veterinary units in which bluetongue outbreaks were declared in livestock in 2007 are shown (grey areas).

The geographical coordinates of the sampled sites were recorded using a hand-held GPS receiver and this information was transferred to UTM 10×10 km square (n = 263, see Figure 1) which was the territorial unit – locality – used in this study.

### Predictor variables

To identify the factors that affected *C. imicola* abundance in each square, we performed spatial modelling to compare the observed abundance of the species with 21 explanatory ecogeographical variables related to the following factors (see Table 1): spatial situation (2 variables), topoclimatic conditions (7 variables), hosts (5 variables), and soil (7 variables). These variables were chosen on the basis of their availability at our study scale and their potential predictive power according to previous knowledge on *C. imicola* ecology [16], [18], [19],[34],[35].

**Table 1:** Variables used to model the abundance of bluetongue vector *Culicoides imicola*.

Code	Variable description	Factor	
LO	Longitude (m)	Spatial location	
LA	Latitude (m)		
A	Mean altitude (masl)	*Topography*	Topoclimate
S	Slope (degrees; calculated from *A*)		
T	Mean temperature in May–October (°C)	*Climate*	
Ts	Temperature seasonality		
P	Precipitation in May–October (mm)		
Ps	Precipitation seasonality		
R	Annual radiation (Kwh m^−2^ day^−1^)		
Fred	Favourability for red deer	*Wild hosts*	Hosts
Froe	Favourability for roe deer		
Fwild	Favourability for wild boar		
DL	Livestock density sheep/goat/cattle (ind/Ha)	*Livestock*	
DC	Cattle density (ind/Ha)		
WL	Woodland (%)	Soil	
IL	Irrigated land (%)		
UL	Sparsely vegetated areas (%)		
SL	Scrublands (%)		
NDVI	Normalized difference vegetation index (NDVI)		
NDVIs	NDVI seasonality		
SP	Soil permeability		

The spatial resolution of the predictors was not homogeneous between factors and thus all the ecogeographical information was finally translated into UTM 10×10 km squares using the Extract module of the Idrisi Andes software package.

#### Spatial factors

We included spatial variables (the longitude and latitude of each square's centroid point) in the models to reveal geographical trends in species distribution associated with historical events or species population dynamics [29], .

#### Topoclimatic factors: topography and climate

The importance of the topoclimatic factor in explaining species distribution and abundance at large spatial scales is well known [37], [38]. Thus, we compared the observed abundances with two predictor variables (altitude and slope) that provide orographical information. Altitude was available in digital format by the Land Processes Distributed Active Archive Center (http://LPDAAC.usgs.gov) at a resolution scale of 100×100 m, and slope was calculated based on altitude using the Idrisi SLOPE command [39].

Climate data (temperature and precipitation) were obtained from the Spanish ‘Agencia Estatal de Meteorología’ (AEMET; http://www.aemet.es). These data were created by the regionalization to Spain of the climate change models produced by the Intergovernmental Panel on Climate Change. This study used a general circulation model, CGM2, from the Canadian Climate Centre for Modeling and Analysis. CGM2 was run with the conditions forecast by the Special Report on Emissions Scenarios A2 and B2 [40] for the period 1961–1990 (later projected to future periods). Scenarios A2 and B2 represent an intermediate position of the range of projected temperature change scenarios for Spain, A2 being medium-high and B2 medium-low [41]. So, A2 is defined as a world of strengthening regional cultural identities, with an emphasis on family values and local traditions, high population growth, and less concern for rapid economic development. In turn, B2 is defined as a world in which the emphasis is on local solutions to economic, social, and environmental sustainability, with lower population growth than A2. The scenario A2 is predicted to change in temperature between periods at a higher rate than the B2 scenario, but in contrast, the precipitation is expected to change in a slightly higher rate in B2 than in A2 [41]. These scenarios are usually selected to study the effect of climate change on species distribution in the Iberian Peninsula [27], [42]. Further details on the peculiarities of each emission scenario and the expected climatic changes in the future can be revised, among others, in [27], [41].

Following the procedure to obtain the climatic variables described by [27], we calculated mean temperature and precipitation, and their seasonalities, for each emission scenario (A2 and B2) and study period (present and future 2011–2040). Mean temperature and precipitation were quantified from May to October only since this period includes the annual peak abundance for *C. imicola* [32], . Seasonality was measured as the variation coefficients of monthly means in a year [16]. Mean solar radiation was also considered as potential predictor of species abundance. Unfortunately, mean solar radiation was not included in the list of variables quantified for each emission scenario, and thus we used the same radiation data for both A2 and B2 emission scenarios.

#### Hosts: wild ungulates and livestock

In addition to livestock, several wild species were described as potential hosts for BTV in Spain [44], [45], [46] and therefore for BTV vectors. Thus, the relative abundances of both wild and domestic hosts were considered to explore their effect in explaining the abundance of *C. imicola*.

Unfortunately, data on wild species abundance at a geographical scale are very scarce. We therefore used the favourability function [47] to obtain the environmental favourability for wildlife, i.e. red deer (*Cervus elaphus*), roe deer (*Capreolus capreolus*) and wild boar (*Sus scrofa*), from presence/absence data, as an index of species abundance. The favourability for a species was significantly related to species abundance as described in a previous study [48]. Briefly, the favourability function is basically a logistic regression that assesses the local variations in presence probability relative to the overall species prevalence (ratio of the number of presences to absences). Using the favourability function, the values for all models are levelled according to the species prevalence in each area [47].

The environmental predictors shown in Table 2 were used to model the environmental favourability for potential wild hosts for *C. imicola*. Wild ungulate distribution data were extracted from the study by [49] and were offered for 10×10 km UTM cells. For each species, we performed a forward-backward stepwise logistic regression procedure to select a subset of significant predictors of the species distribution. Probabilities yielded by logistic regression (*P*) may be used to calculate favourability values (*F*), where *n_1_* is the number of presences and *n_0_* the number of absences [47].

**Table 2:** Variables used to model the environmental favourability for potential wild hosts of *Culicoides imicola*: red deer, roe deer and wild boar.

Variable description	Red deer	Roe deer	Wild boar
Mean annual precipitation –*P*– (mm)(1)	+		+
Maximum precipitation in 24 h –*MP24*– (mm)(1)	+		+
Relative maximum precipitation ( = *MP24*/*P*)			
Mean annual number of days with precipitation ≥0.1 mm(1)		−	
Mean annual number of hail days(1)			
Mean annual number of foggy days(1)	+	+	+
Mean annual potential evapotranspiration –*PET*– (mm)(1)		−	−
Mean annual actual evapotranspiration (mm) ( = min [*P*,*PET*])	+	+	
Mean relative air humidity in January at 07:00 h –*HJN*– (%)(1)	+	+	−
Mean relative air humidity in July at 07:00 h –*HJL*– (%)(1)	−	+	
Annual air humidity range (%) ( = *HJL*-*HJN*)(1)			
Mean temperature in January –*TJN*– (°C)(1)		−	−
Mean temperature in July –*TJL*– (°C)(1)	+		+
Annual temperature range (°C) ( = *TJL*-*TJN*)(1)	+		
Mean annual temperature (°C)(1)		+	
Mean annual number of frost days (minimum temperature ≤0°C)(1)			−
Continentality index(1)	+		
Humidity index(1)	+	+	+
Mean annual insolation (hours year^−1^)(1)		−	
Distance to the nearest town with more than 100,000 inhabitants (km)(2)	+	+	
Distance to the nearest town with more than 500,000 inhabitants (km)(2)	+		
Distance to the nearest highway (km)(2)	+	−	+

Variables included in each model and the sign of their coefficients (positive or negative) are shown. All variables were retained at p<0.01.

Sources:

(1) [30];

(3) [53]; data on the number of inhabitants of urban centres taken from the ‘Instituto Nacional de Estadística’ (http://www.ine.es).



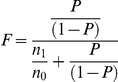



Data on livestock were provided by the Spanish ‘Ministerio de Medio Ambiente y Medio Rural y Marino’ (http://www.marm.es) at a regional veterinary unit level (n = 490 in continental Spain) for 2008. Census data on cattle and small ruminants (sheep and goats) were used to estimate two predictors, livestock density (sheep, goat and cattle) and cattle density. We considered that all domestic ruminants are susceptible to host BTV — and therefore to *C. imicola* — and thus they were together considered in a livestock abundance index which was used in the models. Cattle density was independently considered in the models because of the greater amount of dung production compared to other domestic ruminants. The higher amounts of organic material contributed by cattle may promote breeding sites favourable to *C. imicola* despite the fact that this midge species does not directly breed in cattle dung unlike other *Culicoides* spp. [19]. These variables were transferred from counties to UTM 10×10 km squares, assuming that the mentioned densities were constant through the regional veterinary unit. Therefore, all UTM squares included in a unit — the major part of its surface area — had the same density values.

#### Soil: land cover and pedological variables

Six predictors were included in our model which were related to land cover (including two NDVI-related predictors) and one related to soil permeability (Table 1). NDVI, and its seasonality, were included within this factor, and not within climate [50]; even when it is indirectly related to precipitation, NDVI is a measure of the amount and vigour of vegetation on the land surface directly related to soil moisture [51].

For each UTM square, the frequency of occurrence was calculated of four land cover variables (woodland, irrigated land, sparsely vegetated areas and scrubland) — which were obtained from the CORINE Land Use/Land Cover database [52] — at a spatial resolution of 250×250 m (Table 1). The annual mean value and seasonality (see above for details on the calculations) of the NDVI were derived from a monthly imagery dataset over an 18-year period (from 1982 to 2000) downloaded from the NASA Goddard DAAC website (ftp://daac.gsfc.nasa.gov/data/avhrr/) at a resolution scale of 1000 m. Finally, soil permeability was obtained from a map of synthesis of ground-water aquifers with three different permeability classes [53]. We determined soil permeability for each UTM 10×10 km square by calculating the average of the values assigned to the pixels within the square.

### Statistical analyses

#### Abundance model

Firstly, we avoided correlations between predictor variables related to a specific factor since multicollinearity within a factor unnecessarily affects the automatic stepwise variable selection procedures in regression analysis. Two variables are correlated when the absolute value of Spearman's coefficient is higher than or equal to 0.8 [54]. When two or more explanatory variables were correlated we selected the variable that was most significantly related to the response variable [55] for inclusion in the models.

For each emission scenario we related the observed *C. imicola* abundance (response variable) to the distribution of the climatic variables and the other predictors previously described (Table 1). Given the over-dispersion of our data, for modelling purposes we used generalized linear models (GLM) with a negative binomial distribution and a logarithmic link function [56]. Count models, such as Poisson and negative binomial, were successfully used for studying factors affecting population abundance and for conducting population estimates [57], [58]. We estimated the scale parameter (*K*) for the dependent variable [59], with *x* and *s^2^* as the mean and variance of the data: 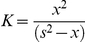



To select a subset of significant predictors we used a forward-backward stepwise model-selection procedure. All steps were assessed to decrease the Akaike Information Criterion, AIC [60].

Finally, we compared the predicted *C. imicola* abundance with the number of BT outbreaks detected in livestock at the regional veterinary unit level (outbreak data were taken from: http://rasve.mapa.es) to assess the spatial association between vector abundance and the BT occurrence rate [50], [61]. For this purpose, five thresholds were fitted to define the highest abundance of *C. imicola* (500, 5000, 12000, 20000 and 50000). We quantified the percentage of localities under each threshold in which at least one BT outbreak was detected in 2007 as an index of spatial overlap between *C. imicola* abundance and the number of outbreaks. Data on BT outbreaks were considered for 2007 only because of the emergence of the highly pathogenic BTV s1 in the Iberian Peninsula and the absence of preventive vaccination of livestock at that time. Thus, the number of BTV outbreaks in 2007 resembled the natural expansion of BTV within the Spanish mainland. This allowed us to associate an epidemiologic meaning to *C. imicola* abundance predictions under scenarios of future climate change. All statistical calculations were made using the SPSS 17 software.

#### Variation partitioning procedure

Variation partitioning is a quantitative method in which the variation in a dependent variable can be separated into independent components reflecting the relative importance of different groups of explanatory variables (factors) and their joint effects. This allows us to specify how much of the variation of the final model is explained by the pure effect of each factor, i.e. not affected by collinearity with other factors in the model, and which proportion is attributable to their shared effect [36], [62], [63].

To do this, independent models for each factor were developed using the statistically significant variables of each factor included in the final model. These partial models are a measure of all the variability explained by each factor (the circles in the Venn diagram). Similarly, we developed partial models for each pair and trio of factors. Then, variation partitioning procedures were applied to the final model output (FMO), i.e. *C. imicola* abundance predicted with all factors. For the partition of 4 factors (*a*, *b*, *c* and *d*), the FMO was correlated against the partial predicted abundance calculated with the retained variables pertaining to 3 of the factors (*a*, *b* and *c*, for example). The residuals of this correlation represent the pure effects of the 4th factor (*d* in this example), i.e. the part of the FMO not explained by the other 3 factors. The amount of variation explained by the pure effect of *d* (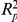) was obtained with the Pearson's coefficient (squared to obtain the amount of variation explained) obtained correlating the FMO with the partial predicted abundance with the 3 included factors as follows: 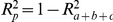. This process was repeated to obtain the variation explained by the pure effect of each factor. The variation explained simultaneously by two factors (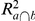; combined effects) was obtained using the partial predicted abundance calculated with the other two factors, specifically with the *R^2^* obtained by correlating FMO and this partial predicted abundance, and the *R^2^* of the pure effects of the two factors involved in the intersection as follows: 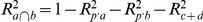. The variation attributable to intersections among trios can be analogously subtracted as follows: 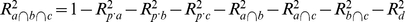 [63]. For applications and further details see [8], [29], [64], [65].

#### Future projections: comparing present and future C. imicola abundances

The predicted abundance of *C. imicola* for each emission scenario was projected to the future by replacing the current temperature and precipitation variables in the models with those expected according to each climate change scenario for the future period. Thus, two predictions of *C. imicola* abundance were forecast, one per emission scenario. To do this, the values of the other variables included in the final models (spatial, topography, host and soil factors) were not modified between periods [27].

Multicollinearity among predictors can be a real problem when a model is projected in other spatial or temporal situations outside the range where it was calibrated [66]. So, we used each predictors' variance inflation factor (VIF) to quantify collinearity among predictors in the models for the present (A2 and B2) because they were projected to a future situation. VIFs were calculated for each predictor as the inverse of the coefficient of non-determination for a regression of that predictor on all others (see [67]). VIF is a positive value representing the overall correlation of each predictor with all others in a model. Previous authors used a value of VIF>10 as the threshold over which multicollinearity can be considered a problem [68], but a more stringent approach is to use values as low as 3 [67].

We used a dual approach to compare present and future *C. imicola* abundances. On the one hand, we quantitatively assessed the relationship between *C. imicola* abundances predicted for the present and abundances forecast for the future. This was performed to assess if the predicted abundances would be higher (or lower) in the future than those predicted for the present at a locality level. Thus, we simply represented in a scatterplot the abundance forecast for the future (y-axis) relative to the abundance predicted for the present (x-axis), and visually evaluated if values for the future were over (or under) the diagonal, which represents the situation where the abundance forecast for the future equals that predicted for the present. On the other hand, we assessed the differences between periods (present and future) in terms of the number of localities with ‘high’ predicted *C. imicola* abundance. To this end we used the same procedure based on *C. imicola* abundance thresholds previously described. We quantified (as percentages) the localities with a predicted abundance over each threshold for the present that were also forecast over this threshold for the future as an index of the localities maintaining high *C. imicola* abundance. We also quantified localities that were forecast to be over the threshold for the future and that were predicted to be under the threshold in the model for the present, which is indicative of the localities where *C. imicola* was forecast to substantially increase its abundance in the future.

## Results

### Wildlife abundance indices

**Figure 2:**
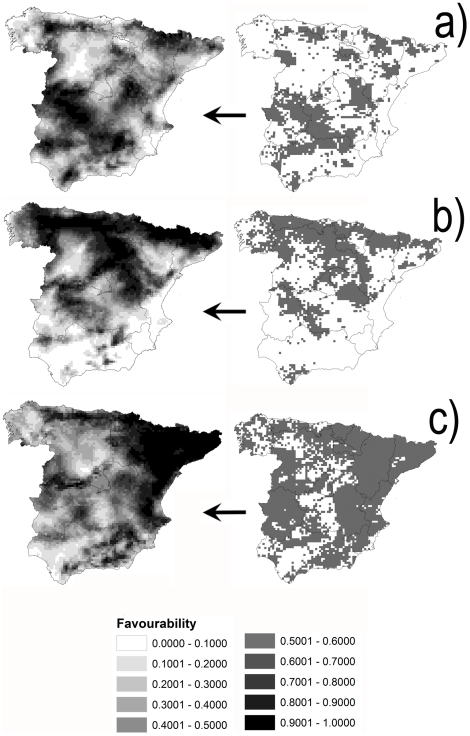
Maps of potential wild hosts abundance. Favourability, where 0 represents minimum favourability and 1 represents maximum favourability, in UTM 10×10 km squares for potential wild hosts of *Culicoides imicola*: red deer (a), roe deer (b) and wild boar (c). Current distributions of these species, referring to 10×10 km UTM grid cells, are depicted in the maps of the right; adapted from [49].

The final functions for wild ungulate favourability models are shown in Table 2. Favourability values for red deer were high across the southwestern and northern areas of peninsular Spain (Figure 2). Higher favourability values were found in the northern half of peninsular Spain for roe deer and in eastern and northeastern areas for wild boar.

### Environmental conditionants of *C. imicola* abundance

The mean observed abundance of *C. imicola*, quantified as the maximum number captured per night between April and October, was (mean ± SE, minimum–maximum): 679.19±200.39, (0–43000) in the period 2005–2008.

**Figure 3:**
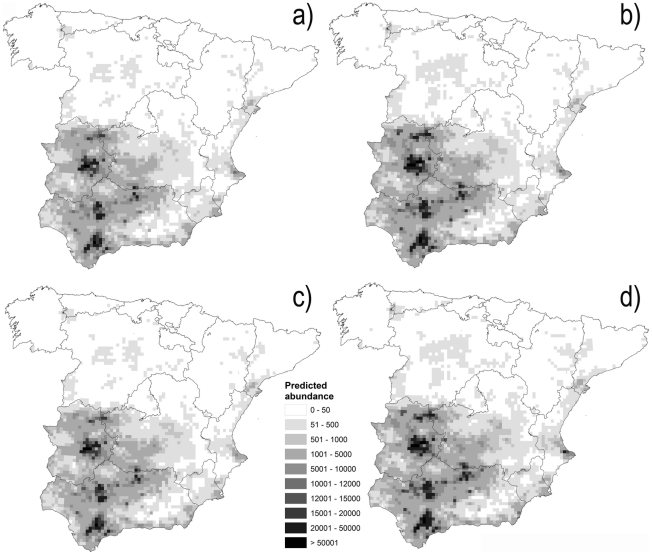
Predicted *Culicoides imicola* abundance. Current predicted *Culicoides imicola* abundance (maximum number of captures per night) according to the CGM2 circulation model and the A2 (a) and B2 (c) emission scenarios (see text for details). Abundance was forecasted for the 2011–2040 period using CGM2 circulation model and the A2 (b) and B2 (d) emission scenarios.

**Figure 4:**
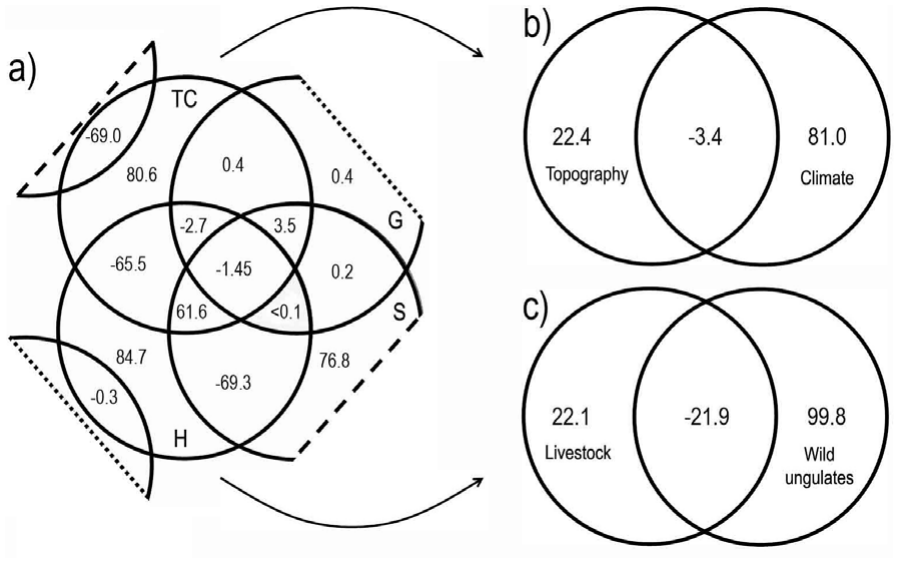
Variation partitioning results. Results of variation partitioning of the final model for the B2 emission scenario (a), and of the partial models obtained for the topoclimatic factor (b), and for the host — wild ungulates and livestock — factor (c). Values shown in the diagrams are the percentages of variation explained exclusively by topoclimate (TC), hosts (H), spatial location (G), and soil (S) and by the combined effect of these factors. See Table 2 for details of the variables included in each of the mentioned factors.

Only 3 out of the 21 considered predictors (latitude, temperature seasonality and annual radiation) were not included in the final A2 and B2 models due to collinearity. The final models for A2 and B2 scenarios explained 59.1% and 59.2% of the total deviance, respectively, and retained variables of the four factors (see Table 3). Table 3 shows the variables which were found to drive *C. imicola* abundance in peninsular Spain. The models obtained for the studied emission scenarios were very similar both in relation to the variables retained and the predicted abundance (Figure 3) regarding the deviation explained. Given the high similarity detected between both final models, we performed the variation partitioning procedure on one of them only. The one selected was B2 since it explained a higher deviance than A2. Variation partitioning demonstrated that the pure effect of host and topoclimate factors explained a high percent (>80%) of the variation (see Figure 4a). The pure effect of soil was the next in importance in explaining the abundance of *C. imicola*. As expected, a high amount of variation was explained by the combined effect of hosts-soil, hosts-topoclimate and soil-topoclimate, since there is a close relationship between each pair of factors, such as the NDVI linking topoclimate and soil. Partitioning complex factors (topoclimate and hosts) into their components (topography/climate and wild/domestic hosts, respectively) demonstrated that within topoclimate the highest amount of variation was explained exclusively by climate (Figure 4b), whereas wild ungulates explained a higher variation than livestock within host factors (Figure 4c).

**Table 3:** Variables included in the *Culicoides imicola* abundance model (GLM binomial negative distribution with logarithmic link function) according to the CGM2 circulation model and the A2 and B2 emission scenarios.

Variable	A2		B2	
	Estimate	Wald	Estimate	Wald
LO	2.11*10^−6^	57.624	1.80*10^−6^	43.931
A	−0.004	1435.267	−0.004	1540.023
S	0.366	824.084	0.369	846.350
T	0.134	42.766	0.147	51.287
P	−0.010	248.338	−0.011	276.241
Ps	−0.010	9.811	−0.017	29.835
Fred	7.551	2273.938	7.619	2314.680
Froe	−2.465	298.214	−2.530	310.008
Fwild	−3.911	871.300	−3.932	882.893
DL	0.258	33.594	0.255	32.732
DC	−1.925	123.697	−1.929	124.670
WL	−0.038	270.870	−0.038	270.036
IL	0.007	23.012	0.007	23.607
UL	0.016	44.732	0.017	52.074
SL	0.010	30.608	0.010	30.282
NDVI	0.033	213.084	0.036	248.979
NDVIs	0.068	232.390	0.069	243.169
SP	1.196	1666.474	1.201	1687.036
Intercept	−2.945	48.666	−2.882	46.331

All variables were retained at p<0.01. Variables coded as in Table 1.

### *C. imicola* abundance and risk of BT outbreaks

A close link was confirmed between *C. imicola* abundance and BT outbreaks, even when the vector abundance predicted for the present was low (Table 4), i.e. over 500 individuals maximum capture. In addition, only around 5% of localities where *C. imicola* abundance was predicted as absent (zero abundance) for the present had at least one BTV outbreak. We should mention that the predicted maximum *C. imicola* abundance of over 500 individuals in the present was almost exclusively restricted to those areas where BT outbreaks took place in 2007 (see Figure 1).

**Table 4:** Spatial overlap between predicted *Culicoides imicola* abundance for present and future periods according to the CGM2 circulation model.

Thresholds/Model		P	FP	BT
500	A2	100	2.72	89.62
	B2	100	4.36	88.93
5000	A2	100	1.67	98.71
	B2	100	2.34	99.13
12000	A2	100	0.79	100
	B2	100	0.98	100
20000	A2	100	0.42	100
	B2	100	0.63	100
50000	A2	100	0.08	100
	B2	100	0.08	100

The A2 and B2 emission scenarios are shown. Different thresholds for the abundance of *C. imicola* were fitted to conduct the estimations. We estimated the percentage of localities with a predicted abundance over each threshold for the present that were also over the same threshold for future periods (P). Additionally, we estimated the percentage of localities predicted over each threshold for the future and under the same threshold in the present model (FP). Similarly, we estimated the percentage of localities with *C. imicola* abundance over the threshold — only with models for the present — with at least one bluetongue outbreak detected in 2007 (BT).

### *C. imicola* abundance under future scenarios of climate change

**Figure 5:**
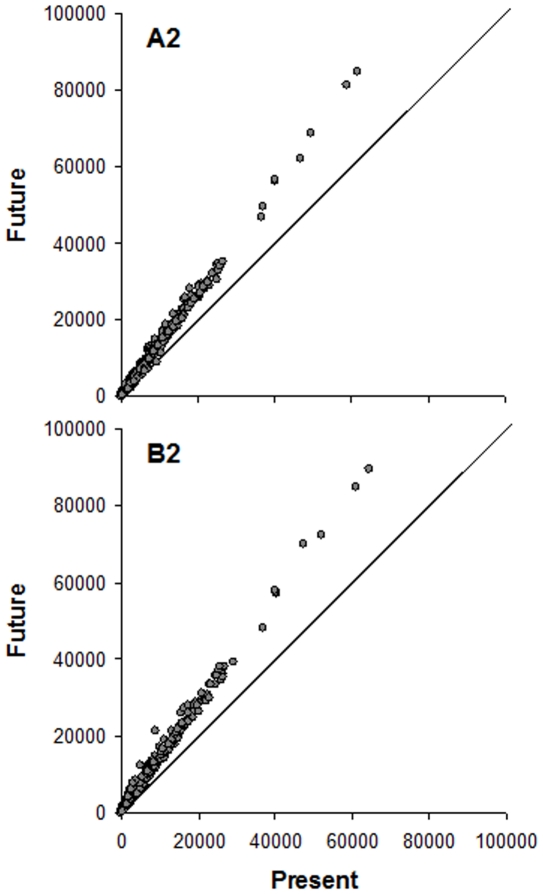
Present/future comparisons in *Culicoides imicola* abundance. Relationships between the predicted *Culicoides imicola* abundance (maximum number of captures per night) for present and future periods according to the CGM2 circulation model and the A2 and B2 emission scenarios.

The projection of the final *C. imicola* abundance models to future climatic scenarios showed an expected increasing total predicted abundance for each locality (Figures 3 and 5) although this increase was not marked. According to the obtained VIF values no relevant effects of multicollinearity are expected in the projections (mean VIF value and range: 2.778, 1.346–4.433; 2.632, 1.345–4.164; for A2 and B2 scenarios, respectively). Our predictions suggested that the distribution area of this vector species will remain quasi-constant in the future (Figure 3, Table 4). Nonetheless, the abundance of *C. imicola* will substantially increase in the localities already occupied. Finally, the higher rates of increases in distribution between periods were obtained for the lower threshold, that is, the increased distribution area is expected to present low abundance of *C. imicola*. The depicted situation was consistent for both emission scenarios; nevertheless, the obtained increment rates were higher for B2 than A2 emission scenarios (Figure 5, Table 4).

## Discussion

### Considerations regarding the methodological approach

We studied the relative contribution of several factors to determine the spatial pattern of abundance of a disease vector arthropod using variation partitioning techniques. This was done with the aim of deepening our understanding of the probable causalities and explanatory powers of predictors in multivariate models, but not with the aim of generating a predictive equation [69]. Thus, it is relevant to enhance the explanatory power of spatial predictive models. The variation partitioning procedure has been widely used to explain the distribution of biodiversity [29], but it has been less frequently applied to epidemiological studies [8], [70].

A large diversity of factors influencing *C. imicola* life-cycle were considered in the present study, and the application of variation partitioning allowed us to determine how much of the variation in the predicted *C. imicola* abundance was explained by the pure effect of each factor (topography and climate, host, soil and spatial factors), and what proportion could only be attributed to their shared effects. To date, only predictors related to climate, topography and soil have been considered for determining the distribution and abundance of *C. imicola* [10], [16], [18], [22], [71]. To the best of our knowledge, this is the first broad assessment of factors determining *C. imicola* — and any vector species — abundance at a large geographical scale. In view of the emergence of the concept of ‘environmental health’ (http://www.oneworldonehealth.org), variation partitioning may help to analyse data on emerging diseases as follows: i) by identifying the most relevant factors determining disease prevalence and spread; and ii) by delimitating epidemiological management units in relation to the factors involved in the transmission of the studied pathogen, e.g. by using freely available data from web-based epidemiological surveillance networks [72].

To date, the effect of climate change on *C. imicola* distribution and abundance only has been assessed in an oversimplified way using idealized scenarios, *sensu* [73], in which an increase in temperature is assumed to be constant for all territorial units in the study area [22]. In this study, changes in climate between periods were considered according to one circulation model and two emission scenarios following the guidelines of the Intergovernmental Panel on Climate Change. In addition, some authors have questioned the validity of models based only on climatic variables for forecasting future species distributions [74], [75], since many factors other than climate play an important role in determining species distributions and their dynamics over time. Consequently, and as conducted in this study, climate effects on species distributions should be considered together with other influential factors to be able to forecast modifications in species ranges due to climate change [27].

### Factors determining *C. imicola* abundance

The models obtained for the two studied emission scenarios were very similar regarding both their explanatory and predictive power. This result agrees with previous studies in which low levels of uncertainty between emission scenarios were reported when modelling species distribution [42], [65], [76]. The climate scenario modelled for the present using the CGM2 circulation model obtained higher precipitations under the A2 emission scenario than under the B2 scenario, although they were quite similar. Differences between scenarios were even less in terms of monthly temperatures. For example, a 1 mm and 0.02°C difference was observed between the A2 and B2 scenarios for the present period relative to annual precipitation and annual mean temperature, respectively [27].

#### Hosts

Variation partitioning showed that the abundance of potential hosts, regardless of the other factors considered, attained the highest explanatory power among the factors considered to predict *C. imicola* abundance. To our knowledge, host abundance has never been considered in predictive modelling for an arthropod vector species, despite hosts being suggested as potential conditioning factors of vector distribution patterns [9], [22]. Additionally, host competence (referring to livestock only) in the epidemiology of BT was recently demonstrated when analyzing factors determining the occurrence of BT outbreaks in Spain [61]. The high explanatory power of wildlife abundance, which is substantially higher than even that attained by livestock density, may be mediated by different explanations as follows: i) wildlife — mainly red deer — may really be playing an important role in the ecology of *C. imicola*, since high densities – up to 69 deer/100 ha [77] – are present in southwestern mainland Spain; and ii) wildlife abundance was modelled with climatic variables, and thus the percentage explained by wildlife can probably be attributed in part to the topoclimate factor.

Unfortunately, the true role of wildlife in explaining *C. imicola* abundance cannot be inferred from our study design, and experimental studies may be necessary. Nonetheless, it should be mentioned that *C. imicola* may feed on wild ungulates, as BTV was detected in these animals [44] in areas where other competent BTV reservoir *Culicoides* spp. are not abundant [16]. Thus, local variations in host availability and composition may impair suitability for *C. imicola* and thus drive its local abundance and local *C. imicola*-borne disease epidemiology. In this respect, and even though our results should be taken with caution, a gap in our knowledge concerning the role of wildlife in *C. imicola* ecology and BTV epidemiology was found and this should be addressed in future studies.

#### Topography and climate

Topography and climate play a relevant role in spatial modelling since the geographic ranges of species at large-spatial scales are limited by abiotic conditions [38]. Thus, our results showed a high explanatory power of the pure effect of the topoclimate factor, mainly due to climatic conditions. Precipitation and its seasonality, and to a lesser extent temperature, were the climatic variables represented in the final models. Our findings contrast with previous studies in which temperature-related variables achieved the highest explanatory power for *C. imicola* distribution models [22], suggesting a degree of temperature-related limitation of vector persistence [35]. The high weight of precipitation found may agree with the requirement of *C. imicola* for humid organically enriched soil as breeding sites [17]. The importance of precipitation over temperature was also reported when modelling BT outbreaks in Spain [61]. Thus, different climatic requirements are probably modulating species distribution and species abundance at a biogeographical scale. Abundance models for *C. imicola* are ecologically (climatically) more similar to BTV distribution models than species distribution models; thus, the former are probably more suitable for consideration in BTV epidemiological studies.

#### Soil

The pure effect of soil (land cover and soil permeability) was the next in importance in explaining *C. imicola* abundance. In our model, the high explanatory power of NDVI, and its seasonality, is consistent with previous studies modelling both *C. imicola* distribution [16], [71], [78] and BT occurrence [50], [61]. Even when NDVI variables were retained in the final models, soil permeability — a variable closely related to the water-holding capacity of soil — was shown to be the most relevant of the soil-related variables [18]. These relationships between NDVI and soil permeability and *C. imicola* abundance can be interpreted from the perspective of the basic requirements of *C. imicola* larvae [79]. Whereas moisture is critical to their survival, nutrients are essential for their development and for the completion of their life-cycle, as mentioned. Thus, *C. imicola* prefers water-saturated, barely permeable, soil with high levels of organic matter, as shown by NDVI values. The land cover variables considered in our study were also retained in the final models, but were less significant than the remaining soil-related variables. This result is consistent with the findings of [20] who reported the limited ability of CORINE classification to accurately predict *Culicoides* breeding in Danish farmland.

#### Spatial components

*C. imicola* abundance was barely explained by the spatial component, demonstrating the absence (or weak presence) of a spatial structure in the abundance data [36]. This factor should be considered in all spatially explicit models in order to reveal geographical trends associated with historical events or species population dynamics [29]. The results obtained can be understood by the high dependence of *C. imicola* on suitable local conditions [10], [17], [18]. To the best of our knowledge, only [61] included the autologistic term in their models on BT epidemiology. Similarly, they found a low degree of spatial correlation, which was attributed to the BT data used in modelling that probably oversimplified the true spatial structure of BTV occurrence.

#### Combined effects

Our results show that a high amount of variation can only be explained by the combined effect of two (or more) factors. Specifically, topoclimate, hosts and soil, in pairs, attained higher amounts of variation. This is due to the interactions between factors and the effects being overlaid subsequently [70]. In this regard, the results obtained were expected since the factors mentioned above are interrelated, such as NDVI linking topoclimate and soil. Variation partitioning or similar tools [80] allow measuring the pure effect of each factor involved in a multi-factorial analysis.

### Projections to future scenarios of climate change

Forecasted projections of *C. imicola* abundance for the near future are only based on changes in precipitation and temperature according to the Intergovernmental Panel on Climate Change [40]. However, indirect effects of climate on land use, host distribution or host population dynamics may modulate the life-cycle of *C. imicola* in the future and hence our predictions. Predicting climate change-associated indirect effects on these factors is difficult but they have to be borne in mind when interpreting our predictions on *C. imicola* abundance.

The projection of the final models to future climatic scenarios showed that the forecast *C. imicola* abundance is expected to increase in each locality (Figure 5), whereas its forecast distribution area will increase by a smaller amount (increase rate less than 4.5%). A stable trend in the *C. imicola* distribution range was recently reported using field data from surveillance programmes, such as those conducted in Portugal [81] and in Italy [26]. *C. imicola* has not appeared to increase its distribution range in Portugal since the mid-1990s, and the results from Italy demonstrated no detectable species range expansion between 2002 and 2007. However, it has been suggested that *C. imicola* is undergoing range expansion in the Mediterranean region, based on field data and on modelling [22], demonstrating a contrasting species response to changing climatic conditions. Other authors concluded that these regional differences are probably related to climatic characteristics [82]. Thus, this species has mainly expanded into warm areas (eastern Spain, northern Italy, southern France and northeastern parts of Greece), whereas areas where temperatures have remained largely unchanged, such as Portugal, have not experienced this type of expansion.

Several hypotheses have been proposed to explain *C. imicola* range stability [26], some of which aid in interpreting the results obtained in this study. The first hypothesis is related to the fact that *C. imicola* may be expanding its distribution ranges at rates which were too low to be detected during our study periods. If the models obtained are projected to more future periods, then significant species expansions will probably be detected. However, the accuracy of the predictions will be reduced, and thus their applicability to disease risk management policy, due to the current uncertainty associated with circulation models and emission scenarios [42]. In addition, a clear increase in abundance was forecast, demonstrating an effective response of the species to climate change between the study periods. Thus, some evidence suggests that factors other than climatic ones may be involved [9], [83].

In this sense, an alternative hypothesis emerges. As previously stated, several factors play an important role in explaining vector and host dynamics over time [75]. Thus, they could determine species ranges in future scenarios [27], with subsequent implications for pathogen emergence and spread [84]. A broad assessment, as conducted in this study, suggests that *C. imicola* may spread, but probably only into those areas where other requirements are fulfilled, rather than moving along a wide front of increased temperature, as suggested by [22].

### Relationships between vector abundance and BTV

Although our study focussed on the factors driving *C. imicola* abundance, our main aim was epidemiological and centred on the study of the determinants of orbiviruses threatening animal health in Europe. Our findings on *C. imicola* abundance suggest that the geographic distribution of orbiviruses expected in future scenarios would not increase if *C. imicola* was the only, or at least the most relevant, competent reservoir of orbiviruses. Nonetheless, recent evidence shows that the ecology of orbiviruses in Europe is more complex than previously thought due to other *Culicoides* spp. acting as new competent vectors [13], [15]. We suggest that a higher *C. imicola* abundance may significantly change the rate of transmission of orbiviruses and facilitate more severe epidemics. It is nonetheless essential to conduct specific studies on the epidemiologic factors driving orbivirus circulation rates, including the influence of competent vectors, before being able to accurately forecast future epidemics.
